# Intact Transition Epitope Mapping—Force Interferences by Variable Extensions (ITEM-FIVE)

**DOI:** 10.3390/biom14040454

**Published:** 2024-04-08

**Authors:** Cornelia Koy, Claudia Röwer, Hans-Jürgen Thiesen, Andrei Neamtu, Michael O. Glocker

**Affiliations:** 1 Proteome Center Rostock, Medical Faculty and Natural Science Faculty, University of Rostock Schillingallee 69, 18057 Rostock, Germany; cornelia.koy@uni-rostock.de (C.K.);; 2 Institute for Immunology, Medical Faculty, University of Rostock, Schillingallee 70, 18057 Rostock, Germany; hj.thiesen@indymed.de; 3 Department of Physiology, “Gr. T. Popa” University of Medicine and Pharmacy, Str. Universitatii nr. 16, 700115 Iasi, Romania; 4 TRANSCEND Centre, Regional Institute of Oncology (IRO) Iasi, Str. General Henri Mathias Berthelot, Nr. 2–4, 700483 Iasi, Romania

**Keywords:** ESI-MS, ITEM mass spectrometry, bio-computation, molecular dynamics, foldon, non-covalent complex, binding strength, intrinsically disordered regions

## Abstract

Investigations on binding strength differences of non-covalent protein complex components were performed by mass spectrometry. T4 fibritin foldon (T4Ff) is a well-studied miniprotein, which together with its biotinylated version served as model system to represent a compactly folded protein to which an Intrinsically Disordered Region (IDR) was attached. The apparent enthalpies of the gas phase dissociation reactions of the homo-trimeric foldon F-F-F and of the homo-trimeric triply biotinylated foldon bF-bF-bF have been determined to be rather similar (3.32 kJ/mol and 3.85 kJ/mol) but quite distinct from those of the singly and doubly biotinylated hetero-trimers F-F-bF and F-bF-bF (1.86 kJ/mol and 1.08 kJ/mol). Molecular dynamics simulations suggest that the ground states of the (biotinylated) T4Ff trimers are highly symmetric and well comparable to each other, indicating that the energy levels of all four (biotinylated) T4Ff trimer ground states are nearly indistinguishable. The experimentally determined differences and/or similarities in enthalpies of the complex dissociation reactions are explained by entropic spring effects, which are noticeable in the T4Ff hetero-trimers but not in the T4Ff homo-trimers. A lowering of the transition state energy levels of the T4Ff hetero-trimers seems likely because the biotin moieties, mimicking intrinsically disordered regions (IDRs), induced asymmetries in the transition states of the biotinylated T4Ff hetero-trimers. This transition state energy level lowering effect is absent in the T4Ff homo-trimer, as well as in the triply biotinylated T4Ff homo-trimer. In the latter, the IDR-associated entropic spring effects on complex stability cancel each other out. ITEM-FIVE enabled semi-quantitative determination of energy differences of complex dissociation reactions, whose differences were modulated by IDRs attached to compactly folded proteins.

## 1. Introduction

Protein function is known to manifest itself predominantly through compactly folded domain structures [1], and functional modulation is in many cases caused by nearby-located intrinsically disordered regions (IDRs) [2,3]. For example, IDRs have been found to flank active site-containing domains in enzymes, such as kinases [4], thereby providing allosteric regulation of enzymatic activity. More specifically, IDRs were considered responsible for the physical tethering of kinases to their substrates [5]. The intrinsic disorder of C-terminal tails of the epidermal growth factor receptor (EGFR) and their extended conformations have been described as important for increasing the capture radius and for reducing the thermodynamic barriers for binding of downstream signaling proteins [6], which stands in line with the protein function modulation concept outlined above. Likewise, it was reported that the IDR of UDP-α-D-glucose-6-dehydrogenase (UGDH) shifted the conformational ensemble to favor inhibitor binding [7]. Consequently, UGDH’s affinity enhancement could be accurately predicted based on the length of the intrinsically disordered segment and was consistent with the entropic force which was generated by an unstructured peptide attached to the protein’s surface.

In our studies, we focus on method development with suitable model substances for determining binding strengths between components of non-covalent complexes. Variants of complex components, which consist of closely related molecules, are distinguishable by their atomic composition, and in cases where hetero-oligomers are produced, they allow investigating whether or not induced variations also cause changes in physico-chemical properties, such as complex stability. T4 fibritin foldon (T4Ff) is a miniprotein of 27 amino acids with a strong tendency to trimerize in a simple fold comprising a β-hairpin that is preceded by a type-II polyproline helix. Folding kinetics has been studied and T4Ff has been considered as evolutionary optimized for fast and efficient trimerization of fibritin [8]. In addition, the T4Ff miniprotein has often been used in biophysical studies, in cooperative protein folding and oligomerization studies [9], and in analyzing the structural features of similar fibrous proteins fused to the foldon domain [10]. By covalently adding chemically defined spacer groups with an attached biotin moiety to T4Ff, we generated a well-defined model system consisting of a compactly folded miniprotein with an extended IDR tail which, nevertheless, maintained its capability to trimerize via non-covalent protein–protein interactions. To investigate the mutual influences of IDRs on complex stability by mass spectrometry, we examined dissociation reactions to determine apparent kinetic and quasi-thermodynamic values in the gas phase of multiply protonated ions from (i) T4Ff homo-trimers (F-F-F) and (ii) biotinylated T4Ff homo-trimers (bF-bF-bF), as well as from (iii) singly biotinylated T4Ff hetero-trimers (F-F-bF) and (iv) doubly biotinylated T4Ff hetero-trimers (F-bF-bF).

Mass spectrometry-based methods for non-covalent complex binding strength analysis, such as Intact Transition Epitope Mapping–Thermodynamic Weak-force Order (ITEM-TWO) [11,12,13], have proven to be able to determine energy differences of protein complex dissociation reactions. Electrospray mass spectrometry is applied to determine apparent binding energies and quasi equilibrium dissociation constants of protein complex dissociation reactions in the gas phase. The mixing of solutions with interacting (monomeric) proteins initiates *in-solution* protein complex formation. The complexes’ binding strengths are determined in the gas phase after electrospraying the protein complex-containing mixture and subsequent mass spectrometric isolation of complex ions. Ion intensities of the complex and of dissociated products are recorded under different collision-induced dissociation (CID) conditions.

Additionally, with Intact Transition Epitope Mapping–Force Differences between Original and Unusual Residues (ITEM-FOUR), a method for studying single amino acid polymorphism (SAP)-related changes in molecular interactions, the influences of specific amino acid residue exchanges on alterations in the binding strength of protein complexes were investigated and compared to *in-solution* ITC-derived data [14]. To specify the bioanalytical procedure described here for investigating compactly folded proteins with attached IDRs, and to differentiate it from previously developed methods, we named our approach “Intact Transition Epitope Mapping—Force Interferences by Variable Extensions (ITEM-FIVE)”.

## 2. Materials and Methods

### 2.1. Preparation of Foldon and Biotinylated Foldon Solutions

T4Ff (27 amino acids, aa sequence GYIPEAPRDGQAYVRKDGEWVLLSTFL-amide, C_142_H_214_N_36_O_41_, MM_monoiso_: 3079.58 Da, MM_avg_: 3081.48 Da) and biotinylated T4Ff (Btn-ado-ado-GYIPEAPRDGQAYVRKDGEWVLLSTFL-amide, C_164_H_251_N_41_O_36_S, MM_monoiso_: 3594.82 Da, MM_avg_: 3597.11 Da were obtained from BIOSYNTHAN (Gesellschaft für bioorganische Synthese mbH Berlin, Germany, https://www.biosynthan.de, accessed on 1 October 2020) as lyophilized TFA salts. Powders were stored at −20 °C until further use. For preparation of stock solutions, 100 µg of the respective lyophilized powder were dissolved in either 100 µL of 50 mM ammonium acetate, pH 6.9, or in 10% acetic acid mixed with methanol (9:1, *v*/*v*), pH 2.0. Solutions were stored at −20 °C until further use.

### 2.2. Desalting of Biotinylated Foldon Solution

Desalting of the biotinylated T4Ff stock solution was performed as described previously [15,16]. In brief, two layers of C18 material (Empore C18 Extraction Disc, Model 2215 3M, Saint Paul, MN, USA) were placed in a 200 μL pipette tip. The C18 filter pieces were then conditioned with 50 μL methanol, followed by 50 μL of a solution consisting of 80% acetonitrile and 20% aqueous acetic acid (0.5%). Centrifugation was performed at 1000 rpm (MiniSpin, Eppendorf, Hamburg, Germany) for three minutes. Subsequently, 10 μL of the biotinylated T4Ff-containing stock solution was loaded onto one StageTip together with 17.5 μL of 0.5% acetic acid. Solvents were eluted by centrifugation. Centrifugation was performed as described above and was repeated in the subsequent steps. After washing with 50 µL of 0.5% acetic acid solution (eluate was discarded), the pipette tip was transferred into a fresh reaction tube. Biotinylated T4Ff was then eluted from the C18 material with 20 µL of a solution that consisted of 80% acetonitrile and 20% aqueous acetic acid (0.5%) by centrifugation. The eluate was lyophilized for 10 min in a vacuum concentrator (SpeedDry RVC 2-25 CDplus; Martin Christ GmbH, Osterode, Germany). The lyophilized biotinylated T4Ff was resuspended either in 40 μL of 50 mM ammonium acetate, pH 6.9, or in 40 μL of 10% acetic acid/methanol (9:1, *v*/*v*), respectively. Solutions were stored at −20 °C until further use.

### 2.3. Protein Concentration Determination

Protein concentrations of (biotinylated) T4Ff stock solutions, as well as of working solutions after desalting, or from T4Ff hetero-trimer-containing mixtures, were determined using the Qubit^®^ Protein Assay Kit (Life Technologies Corp., Eugene, OR, USA). For calibration, a Qubit^®^ working solution (Qubit^®^ reagents diluted 1:200 in Qubit^®^ buffer) and three calibration standards with BSA concentrations 0, 200, and 400 ng/μL, respectively, were prepared in a ratio of 20:1, each, and incubated for 15 min at room temperature. The calibration standards were measured using the Qubit^®^ 2.0 fluorometer (Invitrogen AG, Carlsbad, CA, USA). From each T4Ff-containing solution, 4 μL were diluted with 196 μL of the Qubit^®^ working solution. These solutions were incubated for 15 min at room temperature and measured using the Qubit^®^ 2.0 fluorometer. The calculation of the T4Ff concentration was based on the previously established calibration curve [17].

### 2.4. Generation of Mixtures Consisting of Foldon Homo-Trimers, Biotinylated Foldon Homotrimers, and of Hetero-Trimers

Equimolar mixtures of T4Ff homo-trimers and T4Ff hetero-trimers dissolved in 50 mM ammonium acetate, pH 6.9, were prepared by pipetting together the appropriate volumes of the respective (biotinylated) T4Ff homo-trimer-containing (desalted) solutions. Mixtures were incubated for at least one hour at room temperature, with gentle shaking prior to off-line nanoESI-MS analysis [12].

### 2.5. Nanospray Needle Preparation

NanoESI capillaries for off-line measurements were prepared in-house, as previously described [12]. Briefly, borosilicate glass tubes (BF 100-50-10, inner diameter = 0.5 mm, outer diameter = 1.0 mm), were produced using a P-1000 Flaming/Brown^TM^ micropipette puller system (Sutter Instrument, Novato, CA, USA). After pulling the tips, the needles were shortened to approximately 4 cm in length. Subsequently, capillary needles were gold-coated under argon atmosphere utilizing the SCD005 sputter coater (BAL-TEC AG, Wetter/Ruhr, Germany), setting the following parameters: current 20 mA, sputter time duration 150 s, 5 cm working distance of the table to the gold foil target, vacuum 0.05 mbar, and argon gas pressure 0.5 bar.

### 2.6. Off-Line nanoESI-MS Instrument Settings and Data Acquisition Conditions

For off-line nano ESI measurements, 3.2 µL of T4Ff homo-trimer, biotinylated T4Ff homo-trimer, or T4Ff hetero-trimer-containing mixtures were loaded into separate gold-coated nanoESI capillary needles using microloader pipette tips (Eppendorf, Hamburg, Germany). Mass spectra were acquired on a Synapt G2-S instrument (Waters MS-Technologies, Wilmslow, UK). ITEM-TWO measurements [12] were performed with the following instrument settings: capillary voltage: 0.8–1.0 kV, source offset: 30 V, sample cone voltage: 30 V, trap collisions voltage for simple nanoESI-MS analysis: 0–2 V, source temperature: 30–40 °C; trap gas flow: 0.40 mL/min, and cone gas flow: 100 L/h. For ESI-MS/MS analyses, the trap collision voltage was selected such that the ion signal from the previously isolated precursor ion almost completely disappeared. Instrument calibration was performed with a sodium iodide solution with a concentration of 1 mg/mL, dissolved in isopropanol/water (50:50 *v*/*v*). Data acquisition and processing were performed using MassLynx software version 4.1 (Waters MS-Technologies, Wilmslow, UK).

### 2.7. ITEM-FIVE Experiments

All mass spectra were acquired in positive-ion mode [12], choosing a mass-to-charge window of *m/z* 250–4500. At the starting points of the ITEM-TWO experiments, the quadrupole analyzer was set to isolate the respective quintuply charged precursor ion. Two measurement series were acquired, and mass spectra were recorded at the respective collision cell voltage differences (ΔCV), except for the T4Ff hetero-trimer F-bF-bF, for which only one dataset was recorded. The ΔCV steps were 0, 2, 4, 6, 8, 10, 12, 15, 18, 22, 26, 30, 35, 40, 45, 50, 55 V in the first series. The second measurement series covered ΔCV steps 4, 8, 12, 18, 26V, 35, 45, 55 V. At each ΔCV setting, mass spectra were recorded for 1 min. The combined scans for each ΔCV setting were taken to generate average mass spectra using MassLynx software version 4.1 (Waters MS-Technologies, Wilmslow, UK), without smoothing. The Synapt G2-S instrument is capable to control the quadrupole such that one *m*/*z* window (ion gate) can be set at a time, allowing ions that fall within the chosen *m*/*z* window to travel to the collision cell. Since with ITEM-FIVE it is intended to dissociate only one type of (biotinylated) T4Ff trimer ion at a time, simultaneous transmission of (biotinylated) T4Ff trimer ions with different molecular compositions is prevented. To do so, the ion gate is narrowed, such that the respective 5+ ion (or 6+ ion) of a chosen trimer is exclusively collected, i.e., a trimer with defined molecular composition. Mass spectrometry data have been deposited to the ProteomeXchange Consortium via the PRIDE [18] partner repository with the dataset identifier PXD044721. 

### 2.8. Mass Spectral Data Analysis and Calculation of Apparent Kinetic and Quasi-Thermodynamic Values

From each combined mass spectrum heights of all multiply charged ion signals of T4Ff homo-trimers or T4Ff hetero-trimers (educts), dimers, and monomers (products) at all applied ΔCV values were separately determined and normalized. Then, plots of normalized intensities of educts vs. ΔCV values were fitted to Boltzmann curves with regression coefficients of R2 ≥ 0.99, using Origin software (Origin Lab Corporation, Northampton, MA, USA; version 2018b), as described earlier [12]. Since there is only one tangent line per complex, there is only one Arrhenius plot, and only one Gibbs–Helmholtz plot per (biotinylated) trimer. The equations which were applied to calculate the physical quantities, $k_{m0g}^{\#}$, $K_{Dm0g}^{\#}$, ${\Delta G}_{m0g}^{\#}$, ${\Delta H}_{m0g}^{\#}$, and ${T\Delta S}_{m0g}^{\#}$, have been published [11,12,19,20].

### 2.9. In Silico Simulation and Molecular Modeling of Homo-Trimer and Hetero-Trimer Complexes

The molecular dynamics simulation protocol involved the construction of molecular models for both T4Ff homo-trimers and biotinylated T4Ff homo-trimers, which were used to elucidate interactions between biotin (BTN) and spacer chains in relation to the T4Ff monomers, as well as interactions among the T4Ff monomers themselves. The T4Ff homo-trimer structure was taken from the 1RFO.pdb entry in the Protein Data Bank [21]. For model construction, the first conformation from the NMR ensemble in the 1RFO.pdb file was selected. For the biotinylated T4Ff homo-trimer and hetero-trimers, an appropriate number of biotin plus spacer moieties were constructed and attached to the corresponding foldon monomer utilizing the Schrödinger BioLuminate graphical environment (BioLuminate, Schrödinger, LLC, New York, NY, USA, 2021 [22]. The resultant complexes were solvated in cubic simulation boxes, ensuring a minimum distance of 10 Å between any atom of the simulated protein species and the periodic cell boundaries. The OPLS_2005 force field [23] was applied for the T4Ff homo-trimer ions and the biotinylated T4Ff homo-trimer ions, as well as the hetero-trimer ions, while the TIP3P model was selected for water molecules [24]. All MD simulations (F-F-F, F-F-bF, F-bF-bF, and bF-bF-bF) were performed for 500 ns using the Desmond molecular dynamics package [25] in the NPT statistical ensemble, maintaining constant temperature (300 K) and pressure (1 atm) using the Nosé-Hoover chains thermostat [26] and the Martyna-Tobias-Klein barostat [27]. The number of contacts between the BTN moieties and the T4Ff monomer surfaces were calculated using the open-source community-developed PLUMED library [28] version 2.1 [29] and the PLUMED extension from the VMD version 1.9.4 visualization software [30].

## 3. Results

### 3.1. Characterization of T4Ff and Biotinylated T4Ff Starting Materials

Two foldon proteins, T4Ff and biotinylated T4Ff, were subjected to offline nano ESI-MS analysis to verify their structural integrities (for the amino acid sequence of T4Ff and the chemical structure of the biotin moiety, plus two 8-amino-3,6-dioxa-octanoic acid (ado) spacers see Supplemental Figure S1). When either T4Ff or biotinylated T4Ff was dissolved in acidic aqueous solvents, both proteins afforded triply and quadruply protonated pseudo-molecular ions of the foldon monomers, which were recorded with isotopic resolution (Supplemental Figure S2). Mass spectrometric sequencing confirmed the correct amino acid sequence of T4Ff (Supplemental Figure S3).

**Figure 1:**
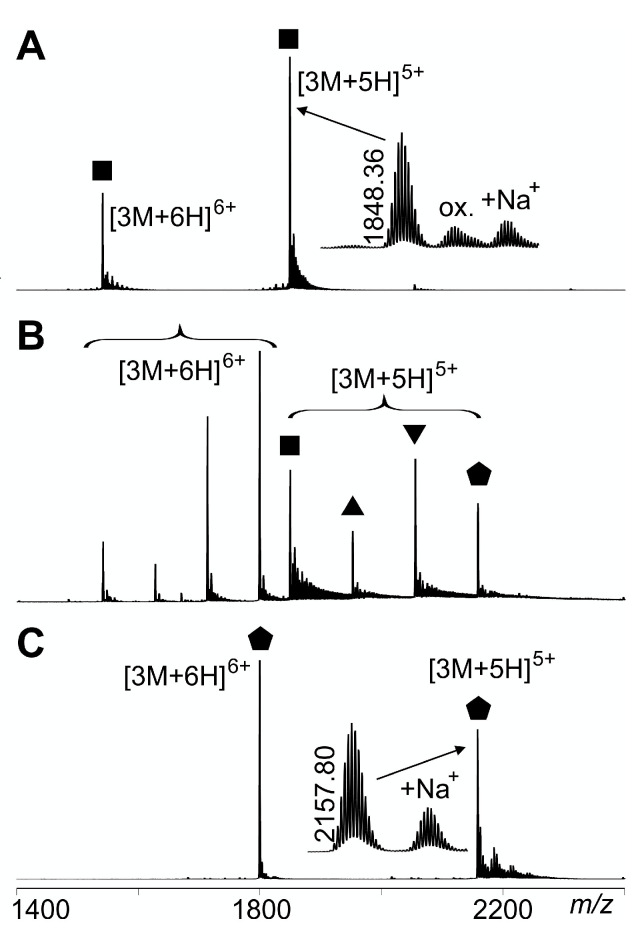
Offline nanoESI mass spectra of multiply charged (biotinylated) T4Ff trimers. (**A**) T4Ff homo-trimer. (**B**) Mixture of (biotinylated) T4Ff hetero-trimers and homo-trimers. (**C**) Biotinylated T4Ff homo-trimer. Protonation states are given. Zooms show isotope patterns of pseudo-molecular ions and *m*/*z* values are given for mono-isotopic ion signals. Partial oxidation (ox.) and sodiation (Na^+^) is indicated. T4Ff homo-trimer: 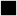; singly biotinylated T4Ff hetero-trimer: 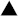; doubly biotinylated T4Ff hetero-trimer: 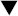; triply biotinylated T4Ff homo-trimer: 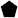. For symbol assignment see Table 1. Solvent: 200 mM ammonium acetate, pH 6.7.

When either T4Ff or biotinylated T4Ff was dissolved in aqueous solvents with neutral pH, both proteins afforded quintuply and hexuply protonated pseudo-molecular ions of the homo-trimers, which, again, were recorded with isotopic resolution (Figure 1). Mixing T4Ff and biotinylated T4Ff solutions provided mixtures of T4Ff and biotinylated T4Ff homo-trimers which were accompanied by singly biotinylated and doubly biotinylated hetero-trimers. The corresponding offline nanoESI mass spectra showed the presence of strong multiply charged pseudo-molecular ion signals for both homo-trimers and both hetero-trimers, whose experimentally determined *m*/*z* values matched well with the calculated values derived from the amino acid sequences and the atom compositions of the respective covalent modifications (Table 1).

Based on the precise molecular mass determinations, the atom numbers of each of the (biotinylated) T4Ff homo-trimers and hetero-trimers were confirmed (Table 1). Knowing the precise atom numbers of the trimers is of importance for calculating temperature dependencies of dissociation reactions (see below).

### 3.2. Dissociation of the (Biotinylated) T4Ff Homo-Trimers and Hetero-Trimers in the Gas Phase

**Figure 2:**
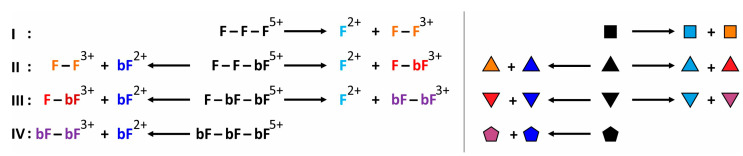
Chemical equations and symbols of dissociation reactions of quintuply protonated (biotinylated) T4Ff trimers. **I**: T4Ff homo-trimer, 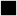. **II**: Singly biotinylated T4Ff hetero-trimer, 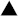. **III**: Doubly biotinylated T4Ff hetero-trimer, 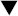. **IV**: Triply biotinylated T4Ff homo-trimer, 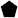. The symbols not only represent the respective product structures but also give information about the educt from which they originate from; for symbol assignments, see also Table 1.

The dissociation of the T4Ff homo-trimer was expected to produce a dimer by release of a foldon monomer (reaction I). Likewise, the dissociation of the triply biotinylated T4Ff homo-trimer was expected to produce a dimer by release of a biotinylated foldon monomer (reaction IV). By contrast, the dissociation of the singly and the doubly biotinylated T4Ff hetero-trimers was expected to produce mixtures of dimers by releasing either foldon monomers (F) or biotinylated foldon monomers (bF; reactions II and III; Figure 2).

**Figure 3:**
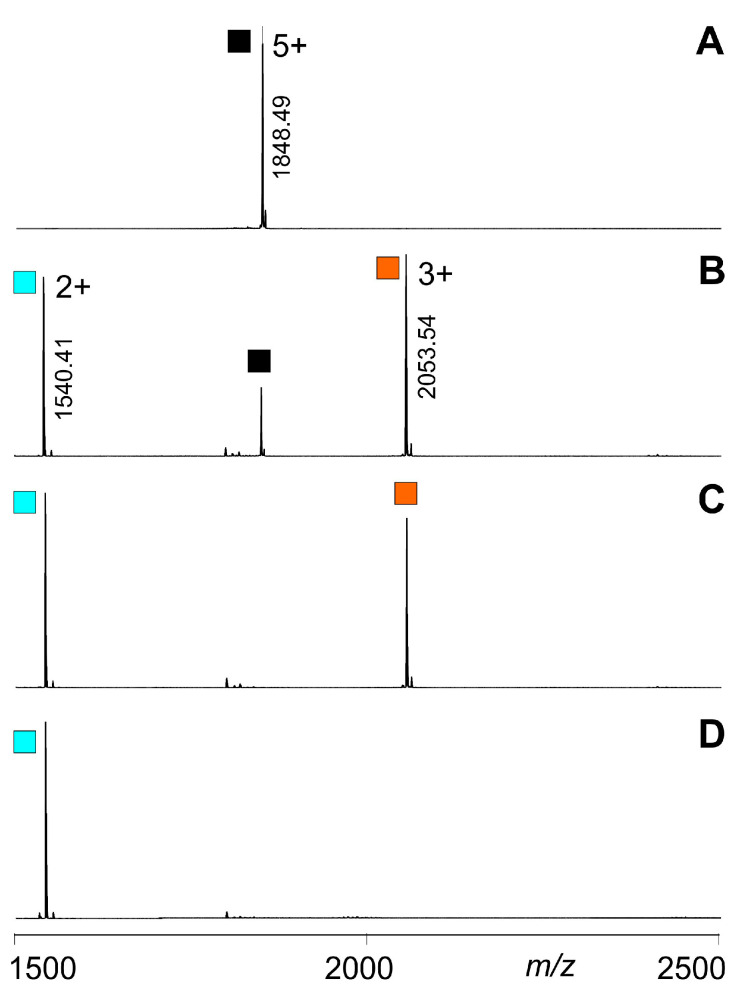
ITEM-TWO analysis of quintuply protonated T4Ff homo-trimers. Different collision cell voltage differences (ΔCV) were applied. (**A**): 0 V. (**B**): 18 V. (**C**): 26 V. (**D**): 45 V. Charge states and *m*/*z* values are given for the T4Ff trimers (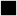) (educts), as well as for the released T4Ff dimers (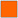) and monomers (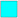) (products). T4Ff concentration was 0.21 µg/µL. For intensities and *m*/*z* values of ion signals, see Supplemental Table S1. For symbol assignments, see Table 1 and Figure 2.

Even though upon hetero-trimer dissociations the mixture complexity of generated products increased, compared to those from homo-trimer dissociations, the courses of relative trimer ion intensities (educts) were feasible to follow in all cases. After selecting and isolating the respective pentuply charged trimer ion signal by mass spectrometric ion filtering, the educt ion intensity diminished upon subsequent increase of ΔCV. At the same time, the product ion intensities increased with higher ΔCV settings (Figure 3 and Supplemental Figures S4–S6).

**Figure 4:**
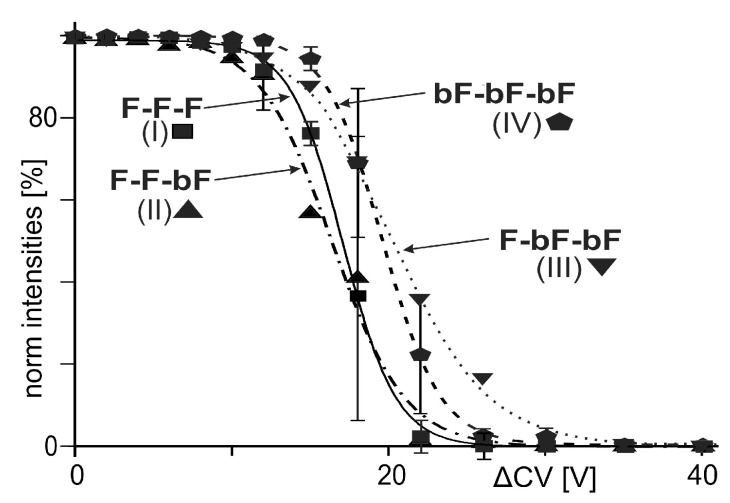
Courses of normalized (biotinylated) T4Ff trimer ion intensities upon gas phase dissociations, plotted as functions of collision cell voltage differences (ΔCV). T4Ff homo-trimer: 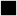; singly biotinylated T4Ff hetero-trimer: 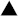; doubly biotinylated T4Ff hetero-trimer: 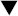; triply biotinylated T4Ff homo-trimer: 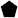. Each data point is the mean of two independent measurements, and standard deviations are shown by vertical bars. Curves were fitted using Boltzmann functions (cf. Table 2). For symbol assignments, see Table 1 and Figure 2.

With the ITEM-FIVE experiments, the focus is on the dissociation processes of the T4Ff trimers, only. Other collision-induced secondary processes, such as protein backbone fragmentations, are not of interest. It turned out that backbone fragmentation happened at rather high collision cell voltage settings (above 60 V), whereas the (biotinylated) T4Ff trimer ion dissociation processes were more or less complete at 40 V (Figure 4).

Ion signal intensities at each ΔCV setting (Supplemental Tables S1–S4) were summed up and set to 100%. Then, normalized educt ion intensities were plotted as functions of ΔCV (Figure 4). The courses of normalized educt ion signal intensities followed sigmoidal shaped curves with Boltzmann characteristics (Table 2).

From the steep parts of the Boltzmann curves, the linear dependencies to ΔCV were calculated, and the tangent lines’ slopes were determined mathematically for all four trimer dissociation reactions (Table 2). The 6+ charge states of the (biotinylated) T4Ff trimers were dissociated as well, and qualitative analysis of the reaction courses provided matching results.

After converting ΔCV dependencies of educts’ ion intensity courses to temperature dependencies, Arrhenius plots (Supplemental Figure S7) and the Gibbs-Helmholtz plots (Supplemental Figure S8) were generated for all four dissociation reactions. Linear extrapolations from the experimentally accessible values to the values at room temperature (ambient temperature) provided the apparent kinetic and the quasi-thermodynamic values ($k_{Dm0g}^{\#}$, $K_{Dm0g}^{\#}$, ${\Delta G}_{m0g}^{\#}$, ${\Delta H}_{m0g}^{\#}$, and ${T_{amb}\Delta S}_{m0g}^{\#}$) of all four trimer dissociation reactions (Table 3). For all (biotinylated) T4Ff trimer dissociation reactions, the **TΔS** terms are large (−62 kJ/mol to −65 kJ/mol) relative to the **ΔH** terms (1.0 kJ/mol to 3.8 kJ/mol), so that **ΔG** in all cases is positive (ca. 65 kJ/mol). This means that the dissociation of the (biotinylated) T4Ff trimer in the gas phase is not spontaneous (it is endergonic, **ΔG** is positive). In addition, the loss of entropy (**ΔS** is negative) upon dissociation is disfavoring the reaction. In the end, this process even consumes energy/enthalpy (it is endothermic, **ΔH** is positive).

Interestingly, the enthalpy values for the biotinylated T4Ff hetero-trimers are distinguishably smaller (1.0 kJ/mol and 1.8 kJ/mol) than those of the (biotinylated) T4Ff homo-trimers (3.3 kJ/mol and 3.8 kJ/mol). And the entropy losses (**TΔS** terms) for the biotinylated T4Ff homo-trimers are distinguishably smaller (−62.3 kJ/mol and −61.8 kJ/mol) than those of the (biotinylated) T4Ff hetero-trimers (−65.3 kJ/mol and −64.2 kJ/mol).

### 3.3. Molecular Dynamics and Entropic Spring Concept

**Figure 5:**
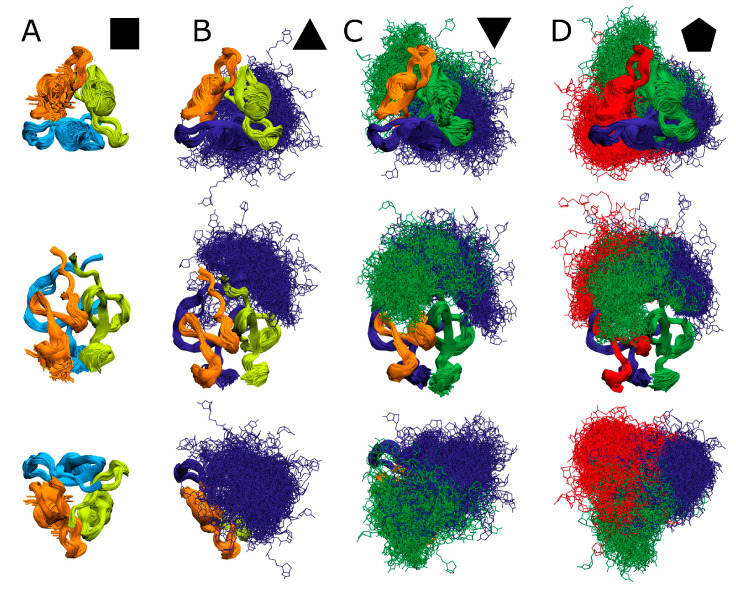
T4Ff structure models of molecular dynamics simulations. Each molecular structure image shows 1000 overlaid T4Ff modeled structures, representing 200 ns of simulation time, each. (**A**): T4Ff homo-trimer (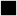). (**B**): Singly biotinylated T4Ff hetero-trimer (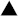). (**C**): Doubly biotinylated T4Ff hetero-trimer (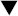). (**D**): Triply biotinylated T4Ff homo-trimer (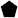). Center panels show side views of molecules, top panels top views, and bottom panels bottom views of (biotinylated) T4Ff trimers. Monomers are color coded. Light color: T4Ff. Dark color: biotinylated T4Ff.

Molecular dynamics simulations of the (biotinylated) T4Ff homo-trimers and hetero-trimers indicated that the protein domains of foldon monomers remained in rather rigid and fairly stable folds in all cases during the entire modelling time courses, each lasting 200 ns (Figure 5). Only the C-terminal residues of each foldon monomer showed wobble, i.e., somewhat greater deviations of their relative positions in space over time.

The atom—atom distances of α carbon atoms from the Ile3 residues of each foldon monomer in a given trimer were determined in all overlaid structure models. These amino acid residues are located near the T4Ff foldon’s N-termini, and, therefore, they are rather closely spaced to the covalently attached biotin moieties. The distances between the Ile3 α carbon atoms were nearly the same among all the T4Ff foldon monomers within a given trimer, with rather few deviations (Table 4).

With increasing numbers of biotins, the distances of α carbon atoms increased slightly. The same is true for α carbon atoms from the Leu23 residues of T4Ff foldon monomers, which are located near the T4Ff foldon’s C-termini. The Leu23 α carbon atoms are further apart from each other, and the deviations in space are larger, which is explained by less stringent structure constraints at the free and wobbling ends of each of the T4Ff foldon monomers.

By contrast, the biotin moieties occupied rather large volumes in space above the T4Ff foldon trimers during the molecular dynamics simulation time intervals indicating very high flexibility. This behavior is reminiscent of IDRs. Interestingly, although the positioning of the biotin moieties seems to exhibit a degree of conformational randomness, they clearly tend to lean towards an adjacent foldon monomer. More specifically, they preferrably contact the clockwise-oriented foldon monomer (Supplemental Table S5).

**Figure 6:**
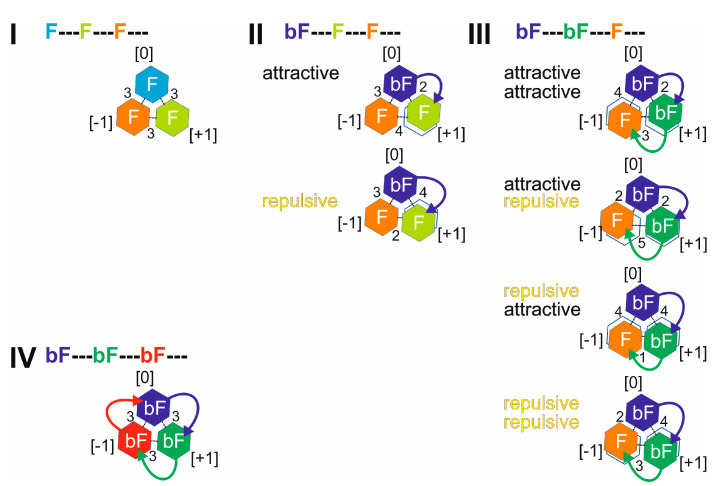
Symmetric and asymmetric transition state geometries of T4Ff trimers. T4Ff homo-trimer (**I**), singly biotinylated T4Ff hetero-trimer (**II**), doubly biotinylated T4Ff hetero-trimer (**III**), and triply biotinylated T4Ff homo-trimer (**IV**). T4Ff monomers are depicted as hexagons and positions are indicated with numbers in brackets ([0]: upper left; [+1]: clockwise oriented from [0]; [−1]: counter-clockwise oriented from [0]). Monomers are color coded. Light color: T4Ff (F). Dark color: biotinylated T4Ff (bF). In symmetric transition states, interfaces are set to be distant by 3 arbitrary units in length (dashed lines). Attractive and repulsive entropic spring effects by covalently bound biotin moieties (arced arrows) cause either shortening or lengthening of interface distances by one unit, each. Then, monomer units are located asymmetrically to each other. Black hexagon frames show T4Ff monomer transition state positions without asymmetric dislocations.

Molecular dynamics simulations reflect ground states with rather high symmetries with respect to foldon monomer assemblies in all four (biotinylated) T4Ff trimers (Figure 6). Therefore, the experimentally determined differences in dissociation energy, which energetically differentiate the dissociation reactions of the hetero-trimers from those of the homo-trimers, are assumed to represent unfolding forces that become effective in the transition states during trimer dissociation (Supplemental Figure S9).

Since the biotin moiety of one of two neighbored foldon monomers behaves like an IDR, we postulate that the biotin-caused transient connections between two T4Ff foldon monomers (the biotin moiety is covalently attached on one side but transiently and non-covalently on the other side) enable attractive or repulsive “entropic spring” effects to become apparent in the T4Ff hetero-trimers. Consequently, due to the resulting asymmetries in the transition states of the biotinylated T4Ff hetero-trimers (induced by the biotin moieties), the transition states experience lowering of their energy levels relative to the energy levels of the transition states of the (biotinylated) T4Ff homo-trimers. Since the ground state energy levels of all four (biotinylated) T4Ff trimers are considered almost indistinguishable from each other, the lowering of the transition state energy levels of the T4Ff hetero-trimers results in lower energy consumption during the dissociation reactions compared to that required for dissociating the T4Ff homo-trimers (cf. Table 3).

## 4. Discussion

Since most bio-macromolecules’ functions include energy difference-related processes, it is of utmost importance to develop and to apply methods that can determine the roles of a biopolymer’s components, as well as of quantifying their (subtle) contributions to a protein’s function and/or its functional modulation. Given the fact that nearly equivalent molecules, including those that form oligomeric structures, share almost the same capacity to combine or react chemically, binding strength differences, induced by the addition of IDRs to compactly folded proteins, can also be assessed by ITEM-FIVE analysis as well.

Differences between intensities of the different charge-states of (biotinylated) T4Ff trimer complexes are very likely due to changes in solvent-accessible surface areas (ΔSASA) of the respective complexes in solution [31]. It has been shown that experimental charge state distribution shifts of ions for protein complexes with different stoichiometries (or different compositions) are explained by relating the magnitude of differences in accessible surface areas (ΔSASA) using a model which is based on the charged residue mechanism. It should be further noted that the dissociation efficiencies of non-covalent complexes depended on their respective charge states [32]. To compensate for this phenomenon, the dissociation reaction properties of the multiply charged (biotinylated) T4Ff trimer ions (which must be considered to carry excess energies) are extrapolated to simulate dissociation processes at ambient temperature without excess energies. This mathematical extrapolation step is one of the main characteristics of the ITEM method, which thereby “eliminates” influences of multiple charges and the acceleration of ions on the dissociation reaction.

Because the dissociation enthalpies of the (biotinylated) T4Ff homo-trimers were comparably high and those of the biotinylated T4Ff hetero-trimers were lower—and the associated entropy values were reciprocally different as well—we searched for likely explanations with a focus on the attached IDR-like biotin moieties. Molecular dynamics simulations indicate that the anchored IDRs contribute negligibly to the formation of the complex in solution. In structural terms, the *in-solution* ground states of all (biotinylated) T4Ff homo-trimers are symmetric. Symmetry is assumed to be preserved upon desolvation and, hence, the ground states of all (biotinylated) T4Ff trimers remain energetically nearly indistinguishable. However, the “entropic spring” concept suggests that the transition states of the biotinylated T4Ff hetero-trimers are asymmetric, whereas the transition states of the (biotinylated) T4Ff homo-trimers remain symmetric.

Entropic springs [33] represent a special category of entropic phenomena, usually described as forces that arise, for example, when a polymer chain or a linear flexible molecule is stretched. In this situation, the number of accessible chain configurations decreases, which in turn creates an entropic force that limits further chain elongation, since every system tends towards higher entropy. Analogous to mechanical springs, entropic forces exhibit a linear relationship with the chain’s end-to-end distance for minor extensions [34]. However, it is important to note that any restriction on chain configurations results in an entropic force, so compressing a molecular chain also gives rise to forces driven by entropy [35,36]. Although the concept of entropic spring originates from polymer physics, which is particularly concerned with elasticity [37,38], this concept has attracted much interest in molecular biology, being applied for explaining fast antibody fragment motion [39], conformational coupling mechanism in the ABC transporters [40], modulation of integrin activation [41], and the force-extension behavior in cardiac titin protein that prevents sarcomere overstretching [42]. Entropic springs have also been identified as functions of intrinsically disordered regions in proteins [43] and were even used to explain more fundamental questions, such as solving the Gibbs paradox [44].

Next, a loss of entropy during (biotinylated) T4Ff trimer dissociation in the gas phase is explained by reducing the degrees of freedom of each of the complex-released (biotinylated) T4Ff monomers/dimers (complex components). There are two concepts that ought to be considered: (1) After dissociation, the T4Ff complex components’ possibilities of assuming positions in space are reduced as compared to the number of possibilities of the positions that the (biotinylated) T4Ff trimer may have been able to take prior to dissociation. In other words, by dividing the (biotinylated) T4Ff trimer into two components (dimer and monomer), the entropy is lowered because there are fewer places the components can be in equiprobably. (2) We postulate that the (biotinylated) T4Ff trimer, with its relatively large surface compared to that of the (biotinylated) T4Ff monomer, provides ample space for the protons that were taken up during the ESI ionization process in the ion source. Protons, which are separated by the largest possible distances because of repulsion, can move around freely and randomly on the molecule’s surface, and this causes that protons to be somewhat depleted on one (biotinylated) T4Ff monomer at times. Then, this allows one (biotinylated) T4Ff monomer, although it is part of the (biotinylated) T4Ff trimer, to also vibrate in a less restrained fashion during those times when proton-dependent charge repulsion is diminished or absent. Hence, despite being bound, the bound (biotinylated) T4Ff monomer may adopt more collapsed conformations (or regions of somewhat more collapsed conformations), which may alternate with more extended conformations (or regions of somewhat more extended conformations) more often over time, as compared to the movements of the free and protonated (biotinylated) T4Ff monomer. A complex-released (biotinylated) T4Ff monomer (at least that fraction which is recorded by mass spectrometry) has taken some of those protons from the (biotinylated) T4Ff trimer upon dissociation. Since the (biotinylated) T4Ff monomer’s surface is smaller than that of the (biotinylated) T4Ff trimer, one expects that there are closer restrictions on the protons’ movements on the (biotinylated) T4Ff monomer. Proton locations then may keep the (biotinylated) T4Ff monomer for longer periods and/or more often in a more extended state to adequately separate the charges. As a result, the released (biotinylated) T4Ff monomer experiences reduced flexibility, as compared to the trimer-bound (biotinylated) T4Ff monomer, which, again, is interpreted as a loss of entropy upon dissociation.

With the data presented here, we answered two questions: whether chemically produced variants of T4Ff monomers maintained the trimer-forming capacities of the original T4Ff, and whether the forces of interactions, as one of the most prominent physico-chemical properties of newly formed T4Ff trimers, were the same or were altered upon chemical modification, i.e., upon the addition of biotin moieties, thereby mimicking IDRs in the vicinity of compactly folded protein domains. Our findings stand in line with known properties of IDRs which enable multivalent, tunable, and malleable molecular recognition that would otherwise be challenging to mediate via folded domains [45]. We exploited the extraordinary trimerization initiation potential of T4Ff which have been previously used for engineering protein needles [46], bio-nanomachines for punctuating host cells [47], and larger self-assembling viroid units [48]. Moreover, T4Ff has been proposed for chemical modification to engineer metal-chelating bipyridine units with a stereo-selective assembly, due to its three-stranded peptide arrangement [49]. Interestingly, during SARS-CoV-2-targeting vaccine development, cloning a foldon sequence into the vaccine target gene was considered a helpful asset in RNA-derived vaccines [50], since it was known that the trimerization of the spike protein induced a significantly higher titer of neutralizing antibodies [51]. The BNT162b1 RNA vaccine candidate, which carried the foldon sequence fused to the spike protein’s receptor binding domain sequence, even entered clinical trials [52,53,54,55], despite the known likelihood that anti-foldon immune reactions would be elicited [56]. Antigenicity is independent of protein folding, and epitopes may be exposed on either compactly folded protein domains [57,58] or on IDRs [59].

Our ITEM data on the dissociation of (biotinylated) T4Ff trimers in the gas phase imply that the opposite reaction, formation of (biotinylated) T4Ff trimers, is spontaneous (exergonic, **ΔG** is negative), releases heat (exotherm, **ΔH** is negative), and is accompanied by a gain of entropy (**ΔS** is positive). In solution, a gain of entropy during (biotinylated) T4Ff trimer formation is explained by the release of ordered solvent (water) molecules from the highly organized solvation spheres of the (biotinylated) T4Ff monomers into the unorganized bulk solution upon complex formation. This effect may even become the driving force. 

As shown, ITEM-FIVE enables the determination, by mass spectrometry, of “apparent” kinetic and “pseudo-thermodynamic” values for dissociation reactions of highly structured non-covalent protein–protein complexes (indicated by the # exponent and by the m0g indexes), as well as IDR-related effects on complex binding strength deviations.

## Figures and Tables

**Table 1:** Molecular information of (biotinylated) T4Ff complexes and T4Ff proteins.

Complex/ Protein	Symbols ^(a)^	*m*/*z* (calcd) ^(b)^	*m*/*z* (exp.) ^(b)^	z	Atom No. ^(c)^
F-F-F	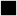	1848.75	1848.49	5+	1299
F-F-bF	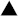	1951.79	1951.52	5+	1371
F-bF-bF	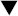	2054.84	2054.54	5+	1443
bF-bF-bF	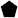	2157.89	2157.78	5+	1515
F-F	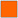 / 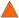	2054.05	2054.29	3+	866
F-bF	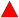 / 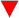	2225.79	2225.25	3+	938
bF-bF	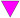 / 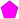	2397.55	2398.04	3+	1010
F	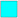 / 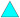 / 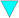	1540.79	1540.41	2+	433
bF	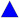 / 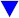 / 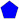	1798.41	1797.98	2+	505

^(a)^ For symbol and color explanations, see Figure 2. ^(b)^ Monoisotopic masses and ^(c)^ elemental compositions derived from the amino acid sequence and of biotin moiety.

**Table 2:** Course characteristics of gas phase dissociations of the trimeric T4Ff complexes.

No.	Complex/ Symbol ^(a)^	Initial [%] ^(b)^	Final [%] ^(c)^	∆CV_50_ [V]	dx [V]	Slope [%/V]	R^2^
I	F-F-F 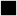	98.78	0.00	17.03	1.70	−14.54	0.999
II	F-F-bF 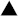	99.64	0.00	16.93	2.32	−10.74	0.995
III	F-bF-bF 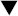	100.00	0.00	20.51	2.95	−8.47	0.999
IV	bF-bF-bF 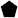	100.00	0.26	19.57	1.84	−13.55	0.999

^(a)^ For symbol explanations, see Figure 2; ^(b)^ Complex amount at lowest applied ∆CV (2 V); ^(c)^ complex amount at highest applied ∆CV (90 V).

**Table 3:** Apparent kinetic and quasi-thermodynamic values for trimeric T4Ff complex dissociation in the gas phase.

No.	Complex/ Symbol ^(a)^	$k_{Dm0g}^{\#}$ [1/s]	$K_{Dm0g}^{\#}$ [Ø] ^(b)^	${\Delta G}_{m0g}^{\#}$ [kJ/mol]	${\Delta H}_{m0g}^{\#}$ [kJ/mol]	${T_{amb}\Delta S}_{m0g}^{\#}$ [kJ/mol] ^(c)^
I	F-F-F 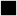	2.17 × 10^7^	3.16 × 10^−12^	65.61	3.32	−62.29
II	F-F-bF 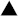	9.16 × 10^8^	3.58 × 10^−12^	65.30	1.86	−63.44
III	F-bF-bF 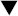	7.83 × 10^8^	3.62 × 10^−12^	65.27	1.08	−64.19
IV	bF-bF-bF 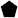	8.68 × 10^6^	3.06 × 10^−12^	65.68	3.85	−61.84

^(a)^ For symbol explanations, see Figure 2; ^(b)^ Unitless number; ^(c)^ T_amb_: 298 K.

**Table 4:** Atom–atom distances of selected amino acid residues in (biotinylated) T4Ff trimer interface regions ^(a)^.

No.	Complex/Symbol ^(b)^	Ile 3		Leu 23	
		Average	Std. Dev.	Average	Std. Dev.
I	F-F-F 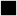	6.18	0.22	9.43	0.50
II	F-F-bF 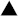	6.20	0.21	9.50	0.49
III	F-bF-bF 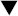	6.26	0.22	9.64	0.51
IV	bF-bF-bF 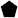	6.33	0.26	9.52	0.52

^(a)^ Numbers in Å; α carbon atom distances; averages from 1000 entries for each measurement; ^(b)^ For symbol explanations, see Figure 2.

## Data Availability

The mass spectrometry data have been deposited to the ProteomeXchange Consortium via the PRIDE [18] partner repository, with the dataset identifier PXD044721.

## Supplementary Materials

The following supporting information can be downloaded at: https://www.mdpi.com/article/10.3390/biom14040454/s1, Figure S1: Structure scheme of biotin anchored to T4Ff; Figure S2: Offline nanoESI mass spectra of foldon monomers; Figure S3: Offline nanoESI tandem mass spectrum of foldon monomer; Figure S4: ITEM-TWO analysis of quintuply protonated singly biotinylated T4Ff hetero-trimer (F-F-bF); Figure S5: ITEM-TWO analysis of quintuply protonated doubly biotinylated T4Ff hetero-trimer (F-bFbF); Figure S6: ITEM-TWO analysis of quintuply protonated biotinylated T4Ff homo-trimers; Figure S7: Arrhenius plot for (biotinylated) T4Ff trimer dissociation reactions in the gas phase; Figure S8: Gibbs-Helmholtz plot for (biotinylated) T4Ff trimer dissociation reactions in the gas phase; Figure S9: Energy diagrams showing the apparent enthalpies of activation required by multiply charged and accelerated (biotinylated) T4Ff trimers; Table S1: Ion intensities, charge states, and *m*/*z* values for the F-F-F homotrimer at various collision cell voltage difference settings; Table S2: Ion intensities, charge states, and *m*/*z* values for the F-F-bF heterotrimer at various collision cell voltage difference settings; Table S3: Ion intensities, charge states, and *m*/*z* values for the F-bF-bF heterotrimer at various collision cell voltage difference settings; Table S4: Ion intensities, charge states, and *m*/*z* values for the bF-bF-bF homotrimer at various collision cell voltage difference settings; Table S5: Biotin contacts with foldon monomer units in T4Ff trimers.
