# Supplementary material for: Intact Transition Epitope Mapping—Force Interferences by Variable Extensions (ITEM-FIVE)
Source: Biomolecules. 2024 Apr 8;14(4):454. doi: 10.3390/biom14040454 (PMC11048379; doi:10.3390/biom14040454)
Supplement: Supplementary file 1 [file biomolecules-14-00454-s001.zip › biomolecules-2916505-supplementary.pdf]

Intact Transition Epitope Mapping – Force Interferences by Variable Extensions (ITEM-FIVE)

Cornelia Koy [1)], Claudia Röwer [1)], Hans-Jürgen Thiesen [2)], Andrei Neamtu [3)], Michael O. Glocker [1)]

1) Proteome Center Rostock, Medical Faculty and Natural Science Faculty, University of Rostock, Schillingallee 69, 18057 Rostock, Germany

2) Institute for Immunology, Medical Faculty and Natural Science Faculty, University of Rostock, Schillingallee 69, 18057 Rostock, Germany

3) TRANSCEND Centre – Regional Institute of Oncology (IRO) Iasi, Str. General Henri Mathias Berthelot, Nr. 2–4 Iași, România; Department of Physiology, Gr. T. Popa University of Medicine and Pharmacy of Iasi, Str. Universitatii nr. 16, Iasi Jud., Romania

Supplement

Supplemental Table S1: Ion intensities, charge states, and m/z values for the F-F-F homotrimer at various collision cell voltage difference settings. **1st determination** (15072021_Foldon_Pep1_MSMS_1849_02.raw)

**ion**	z	**m/z**	**0V**	**2V**	**4V**	**6V**	**8V**	**10V**	**12V**	**15V**	**18V**	**22V**	**26V**	**30V**	**35V**	**40V**	**45V**	**50V**	**55V**
**F-F-F**	**5+**	1848	92850	65322	111678	125873	132104	128171	139805	123661	77073	14291	378	164	44	42	41	9	/
**F-F**	**3+**	2053	204	196	182	321	461	635	1810	7754	22584	54422	52233	59050	29306	5965	1516	198	/
**F**	**2+**	1540	490	498	525	486	1427	3054	7721	31087	95942	238696	258080	357819	421986	441875	399284	585802	/

2nd determination (20072021_Foldon_FBTN_P1_MSMS_1848.raw)

**ion**	z	**m/z**	**0V**	**2V**	**4V**	**6V**	**8V**	**10V**	**12V**	**15V**	**18V**	**22V**	**26V**	**30V**	**35V**	**40V**	**45V**	**50V**	**55V**
**F-F-F**	**5+**	1848	/	/	160145	/	228438	/	223072	/	110400	724	304	/	141	/	115	/	100
**F-F**	**3+**	2053	/	/	334	/	1391	/	5149	/	43963	90997	64632	/	3088	/	80	/	130
**F**	**2+**	1540	/	/	501	/	4032	/	20916	/	172703	410203	577840	/	708641	/	682144	/	376570

Supplemental Table S2: Ion intensities, charge states, and m/z values for the F-F-bF heterotrimer at various collision cell voltage difference settings. **1st determination** (19072021_Foldon_P1BTN_MSMS_1952_01.raw)

**ion**	z	**m/z**	**0V**	**2V**	**4V**	**6V**	**8V**	**10V**	**12V**	**15V**	**18V**	**22V**	**26V**	**30V**	**35V**	**40V**	**45V**	**50V**	**55V**
**F-F-bF**	**5+**	1952	21377	34580	38452	31003	39316	39255	34383	30702	9567	1091	58	41	39	26	20	22	15
**F-F**	**3+**	2054	70	115	119	189	109	256	490	2123	3573	6544	6184	6523	2699	810	252	56	0
**bF**	**2+**	1798	0	218	195	271	259	486	1350	4737	8711	13307	12085	19956	20032	27843	29396	26371	21811
**F-bF**	**3+**	2225	0	0	78	77	87	303	692	1872	4091	5609	5480	5009	2794	978	119	0	0
**F**	**2+**	1541	102	149	251	256	601	1242	3833	14618	31826	47075	43997	65564	63684	78382	79474	81083	65318

2nd determination (20072021_Foldon_FFBTN_P1_MSMS_1951.raw)

**ion**	z	**m/z**	**0V**	**2V**	**4V**	**6V**	**8V**	**10V**	**12V**	**15V**	**18V**	**22V**	**26V**	**30V**	**35V**	**40V**	**45V**	**50V**	**55V**
**F-F-bF**	**5+**	1952	/	/	408760	/	384719	/	403319	/	319882	/	16584	/	266	/	99	/	95
**F-F**	**3+**	2054	/	/	398	/	522	/	1489	/	15830	/	91527	/	74599	/	11741	/	95
**bF**	**2+**	1798	/	/	537	/	1097	/	3474	/	37300	/	234565	/	265857	/	440975	/	477642
**F-bF**	**3+**	2225	/	/	244	/	562	/	1666	/	15678	/	86344	/	76233	/	16104	/	186
**F**	**2+**	1541	/	/	772	/	1644	/	8535	/	100301	/	609293	/	665798	/	913634	/	953036

Supplemental Table S3: Ion intensities, charge states, and m/z values for the F-bF-bF heterotrimer at various collision cell voltage difference settings. **1st determination** (20072021_Foldon_FBTN_P1_MSMS_2055.raw)

ion	z	m/z	0V	2V	4V	6V	8V	10V	12V	15V	18V	22V	26V	30V	35V	40V	45V	50V	55V
**F-bF-bF**	**5+**	2054	/	89313	/	/	84556	79738	75526	72486	65392	60604	51672	5215	2760	860	499	126	235
**bF-bF**	**3+**	2397	/	58	/	/	177	194	388	1050	2173	9035	18432	24319	25440	19185	18473	6062	1361
**F**	**2+**	1541	/	193	/	/	504	777	1308	2775	8512	26937	66681	90641	111032	105951	161977	132230	156167
**F-bF**	**3+**	2225	/	175	/	/	353	416	1094	2614	8423	35251	79991	98470	99147	62855	40741	7406	827
**bF**	**2+**	1798	/	201	/	/	350	711	1292	3444	9399	36182	91335	118962	139491	135419	226755	213665	252786

Supplemental Table S4: Ion intensities, charge states, and m/z values for the bF-bF-bF homotrimer at various collision cell voltage difference settings. **1st determination** (15072021_Foldon_BTN_MSMS_2159_01.raw)

ion	z	m/z	0V	2V	4V	6V	8V	10V	12V	15V	18V	22V	26V	30V	35V	40V	45V	50V	55V
**bF-bF-bF**	**5+**	2158	534373	1411979	1716272	2454268	2253328	2086819	2219942	2401570	1862383	809933	110522	7599	999	222	104	59	29
**bF-bF**	**3+**	2397	85	226	413	653	958	1936	2506	5583	15724	67215	697159	1186044	302551	282999	160255	54076	9182
**bF**	**2+**	1798	0	0	1074	1776	2702	6517	7694	22604	74606	347946	999319	1711191	2170672	2720714	3012266	3512346	3577762

2nd determination (19072021_Foldon_P1FBTN_MSMS_2158.raw)

ion	z	m/z	0V	2V	4V	6V	8V	10V	12V	15V	18V	22V	26V	30V	35V	40V	45V	50V	55V
**bF-bF-bF**	**5+**	2158	19333	58466	58057	55380	62102	74214	55149	52108	30501	6239	479	260	167	124	107	112	32
**bF-bF**	**3+**	2397	0	0	0	0	70	100	179	605	3766	6776	5675	5214	4317	2333	139	140	18
**bF**	**2+**	1798	87	78	133	305	367	580	897	3728	20079	38907	32294	31186	39621	52623	61767	64951	57852

Supplemental Table S5: Biotin contacts with foldon monomer units in T4Ff trimers [a].

	F [b]	bF-bF-bF	F-bF-bF	F-F-bF	F-F-F
in touch with	+1	41.82	63.17	66.10	n.a.
in touch with	0	2.53	2.29	3.12	n.a.
in touch with	-1	2.26	1.00	0.67	n.a.
not in touch	/	62.97	40.12	37.69	n.a.

	F [b]	bF-bF-bF	F-bF-bF	F-F-bF	F-F-F
in touch with	+1	68.47	78.47	n.a.	n.a.
in touch with	0	5.91	6.78	n.a.	n.a.
in touch with	-1	0.50	0.23	n.a.	n.a.
not in touch	/	37.94	28.54	n.a.	n.a.

	F [b]	bF-bF-bF	F-bF-bF	F-F-bF	F-F-F
in touch with	+1	53.24	n.a.	n.a.	n.a.
in touch with	0	1.90	n.a.	n.a.	n.a.
in touch with	-1	0.70	n.a.	n.a.	n.a.
not in touch	/	49.36	n.a.	n.a.	n.a.

a) numbers in %. Color code as in figure 5.
b) foldon unit: 0: foldon unit to which the biotin moiety is bound to; +1: clock-wise oriented foldon unit to the one to which the biotin moiety is bound to; -1: counter-clock-wise oriented foldon unit to the one to which the biotin moiety is bound to
n.a. not applicable

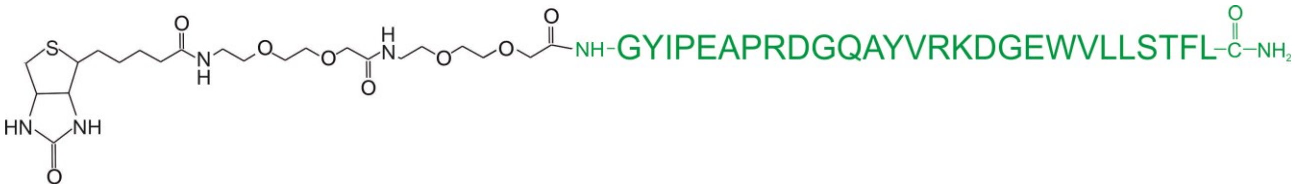

Supplemental Figure S1: Structure scheme of biotin anchored to T4Ff. Biotin is covalently attached to T4Ff's N-terminal amino group via two 8-amino-3,6-dioxa-octanoic acid spacers. T4Ff's amino acid sequence (green) is shown in single letter code and its C-terminal amido group is indicated.

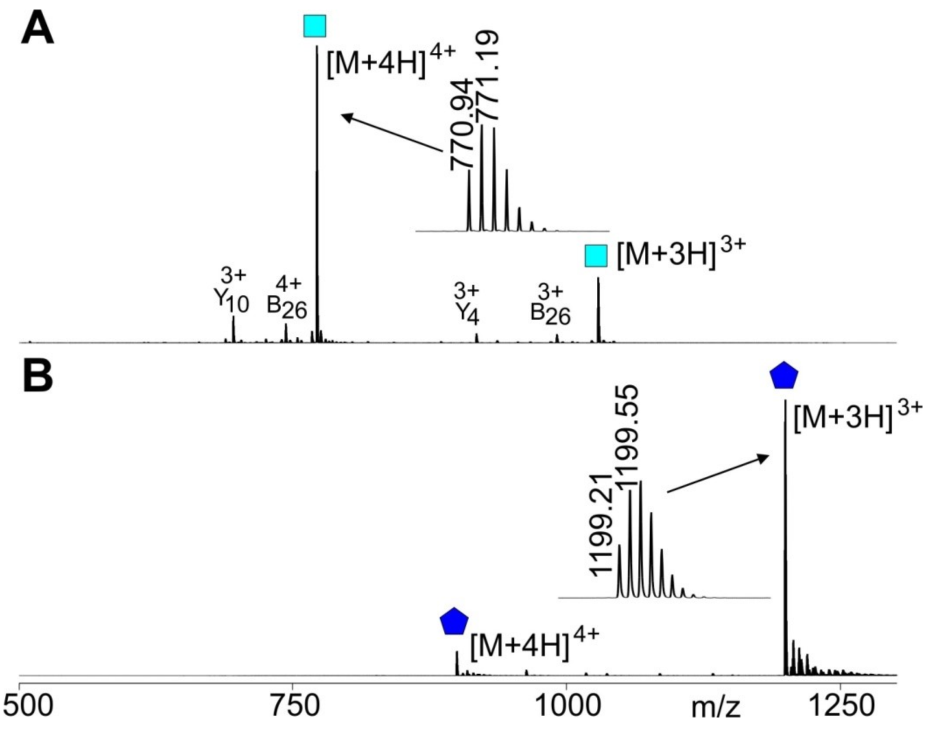

Supplemental Figure S2: Offline nanoESI mass spectra of foldon monomers. **A:** T4Ff ( ). **B:** Biotinylated T4Ff ( ). Protonation states of multiply charged ion signals are given. Zooms show isotope patterns of pseudo-molecular ions and m/z values are given for mono-isotopic ion signals and ion signals with peptides which contain one ^{13}C atom. Fragment ions are labeled. For symbol assignment see Table 1. Solvent: 10% acetic acid / methanol (9:1 v/v), pH 3.

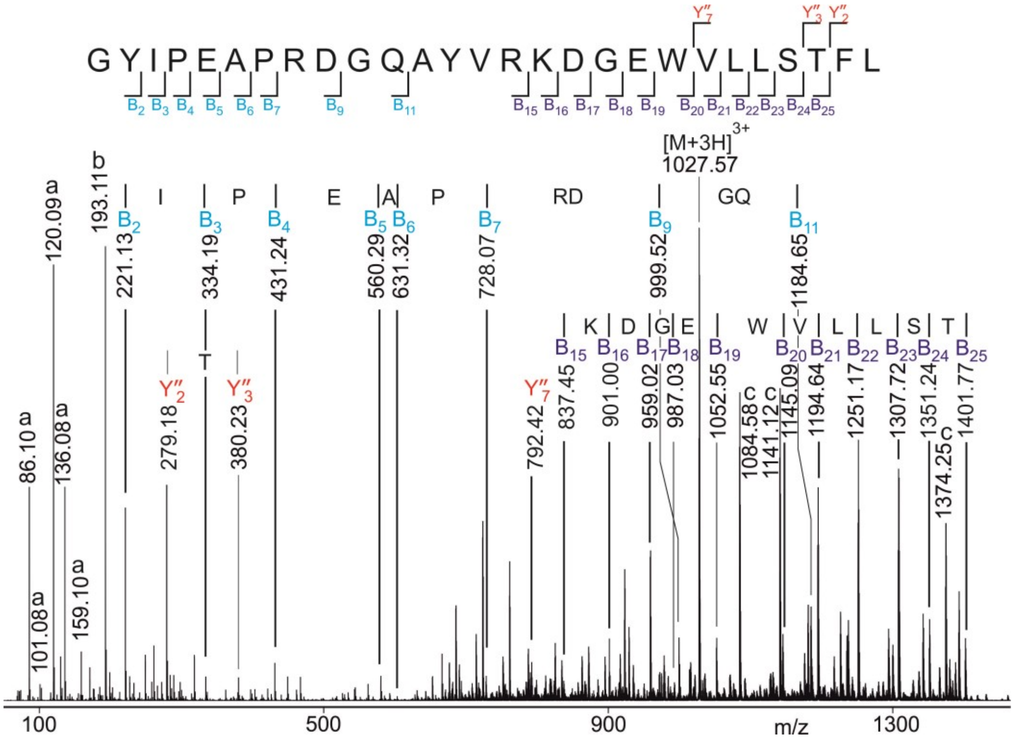

Supplemental Figure S3: Offline nanoESI tandem mass spectrum of foldon monomer. The triply-protonated pseudo-molecular ion of T4Ff served as parent ion. B and Y'' ion signals are labeled and m/z values of selected ion signals are given. Amino acid sequence is shown in single letter code. Solvent: 10% acetic acid / methanol (9:1 v/v), pH 3.

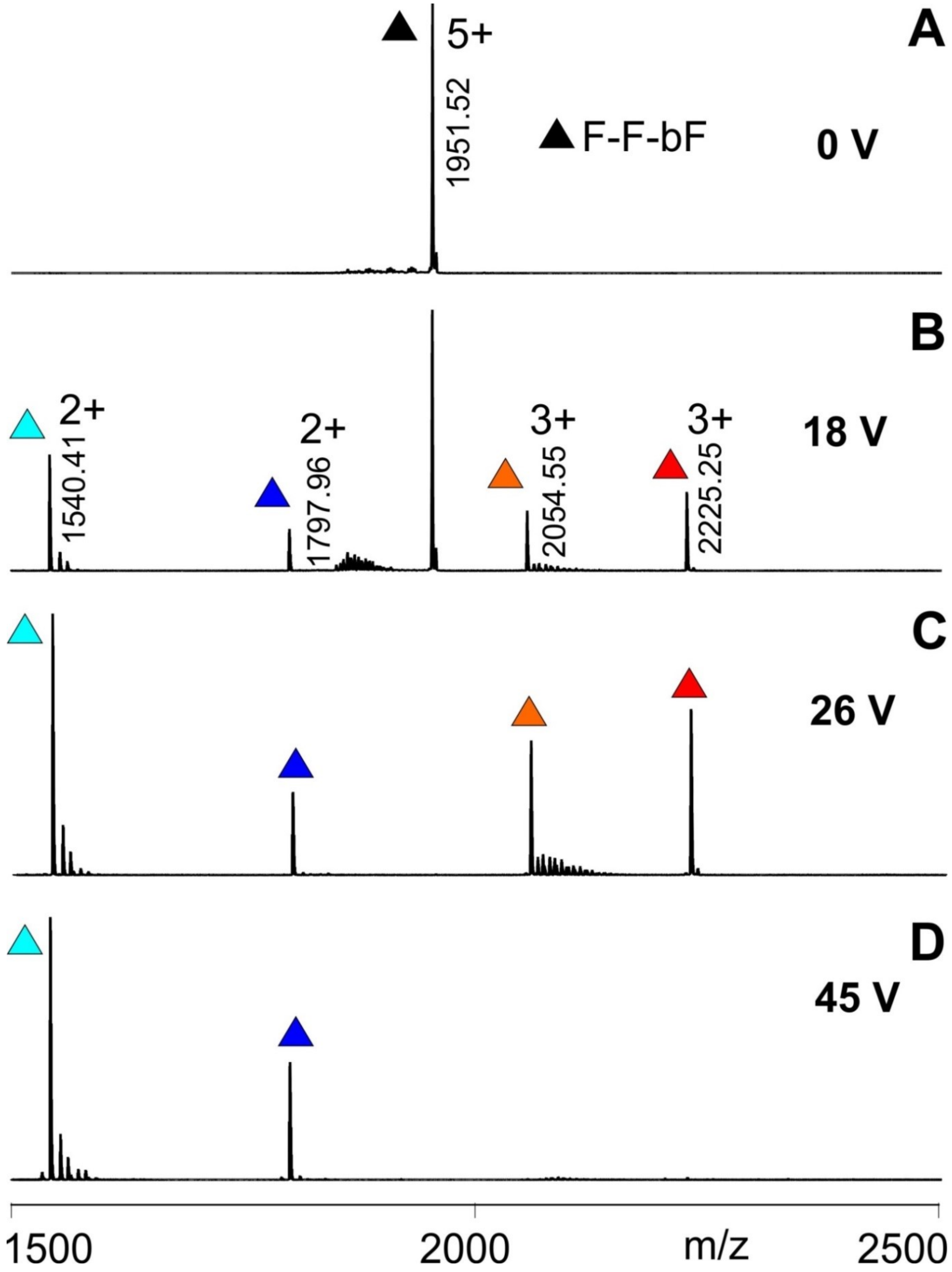

Supplemental Figure S4: ITEM-TWO analysis of quintuply protonated singly biotinylated T4Ff hetero-trimer (F-F-bF). Different collision cell voltage differences (ΔCV) were applied. **A:** 0 V. **B:** 18 V. **C:** 26 V. **D:** 45 V. Charge states and m/z values are given for the singly biotinylated T4Ff trimers (▲) (educts) as well as for the released T4Ff dimers (▲ and ▲) and monomers (△ and ▲) (products). T4Ff concentration was 0.21 µg/µl. For intensities and m/z values of ion signals see Supplemental Table 2. For symbol assignments see Table 1 and Figure 2.

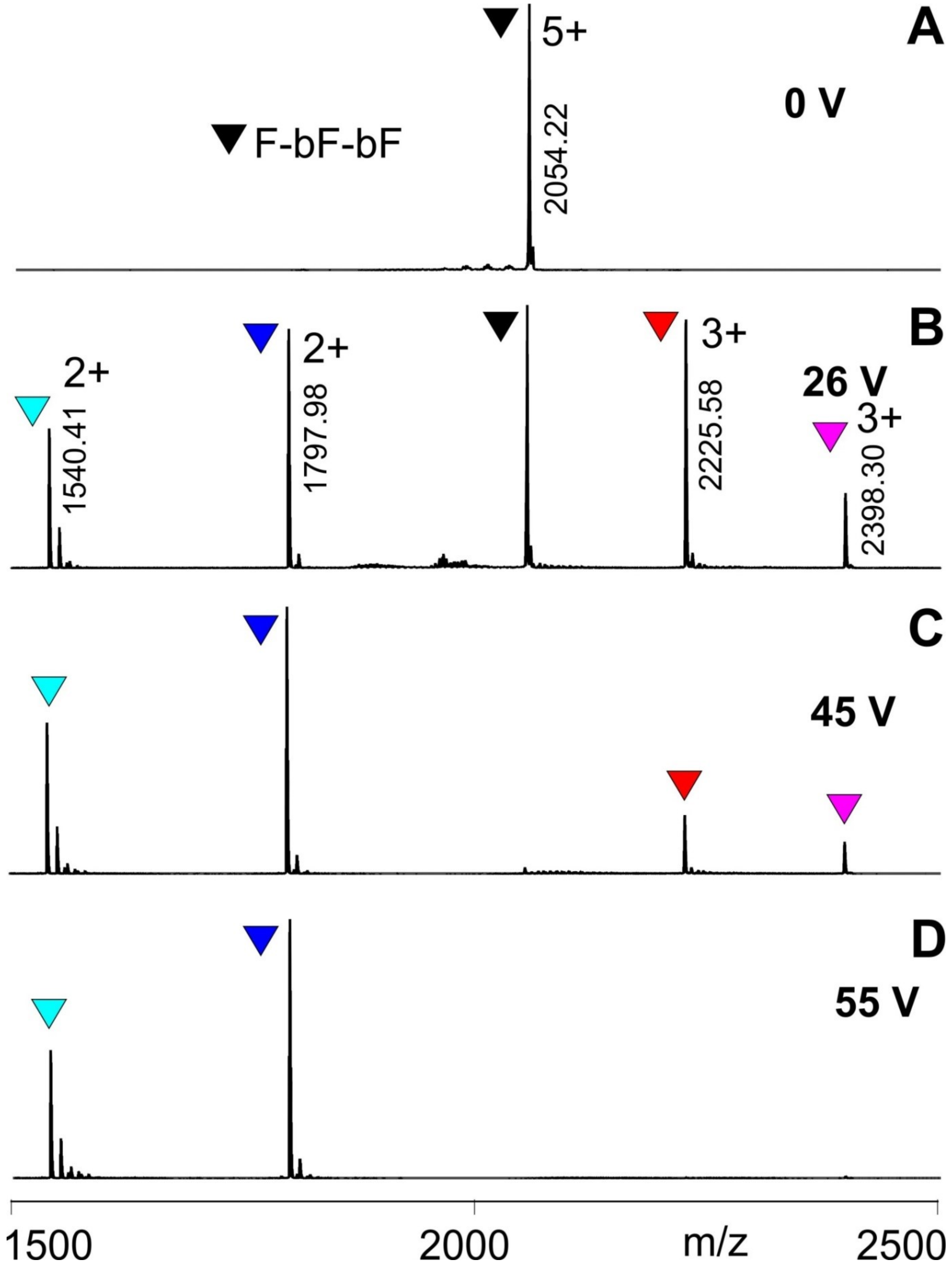

Supplemental Figure S5: ITEM-TWO analysis of quintuply protonated doubly biotinylated T4Ff hetero-trimer (F-bF-bF). Different collision cell voltage differences (ΔCV) were applied. **A:** 0 V. **B:** 26 V. **C:** 45 V. **D:** 55 V. Charge states and m/z values are given for the doubly biotinylated T4Ff trimers (▼) (educts) as well as for the released T4Ff dimers (▼ and ▼) and monomers (▽and▼) (products). T4Ff concentration was 0.21 µg/µl. For intensities and m/z values of ion signals see Supplemental Table 3. For symbol assignment see Table 1 and Figure 2.

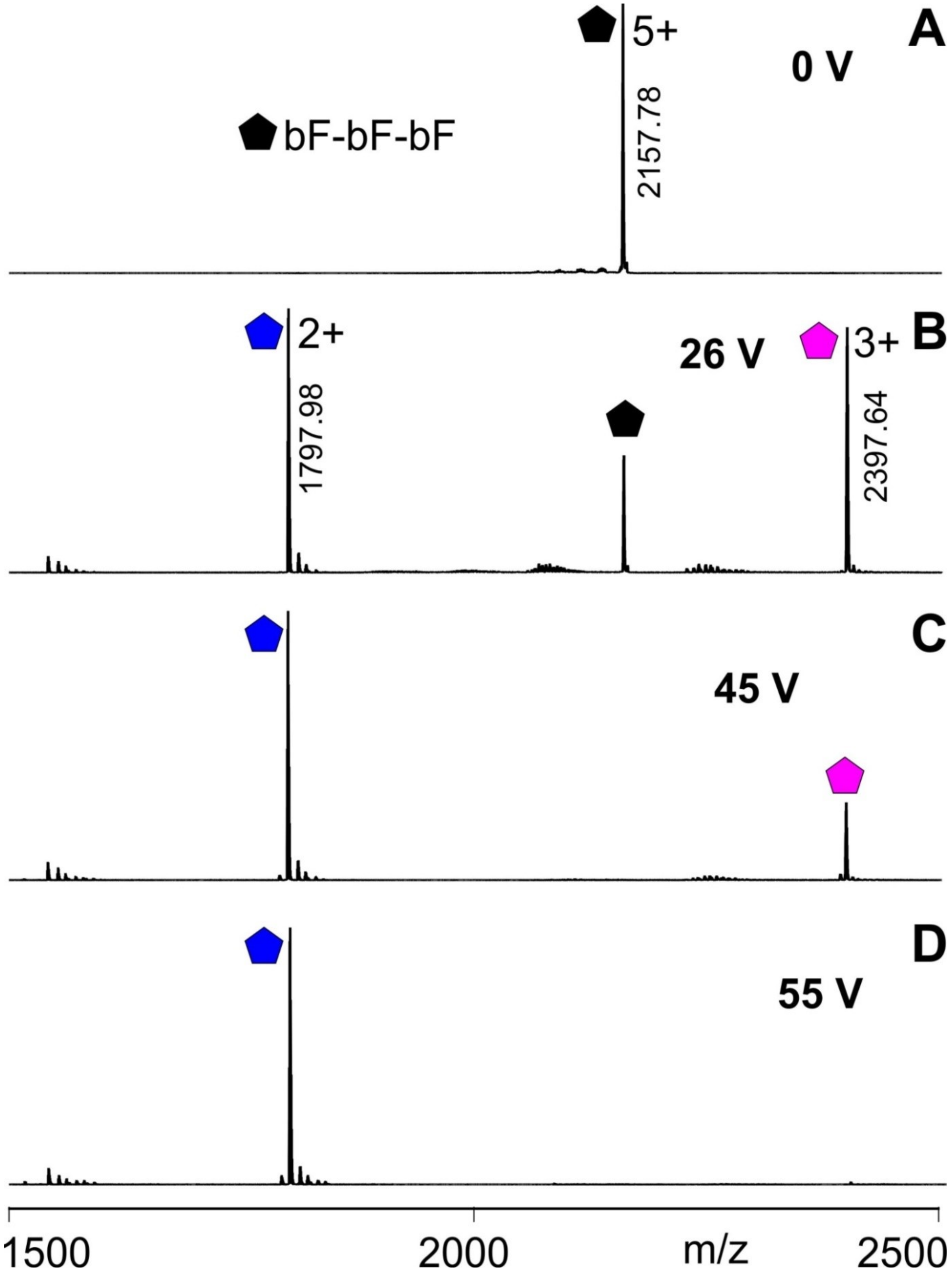

Supplemental Figure S6: ITEM-TWO analysis of quintuply protonated biotinylated T4Ff homo-trimers. Different collision cell voltage differences (ΔCV) were applied. **A:** 0 V. **B:** 26 V. **C:** 45 V. **D:** 55 V. Charge states and m/z values are given for the biotinylated T4Ff trimers (⬟) (educts) as well as for the released biotinylated T4Ff dimers (⬟) and biotinylated monomers (⬟) (products). Biotinylated T4Ff concentration was 0.21 µg/µl. For intensities and m/z values of ion signals see Supplemental Table 4. For symbol assignment see Table 1 and Figure 2.

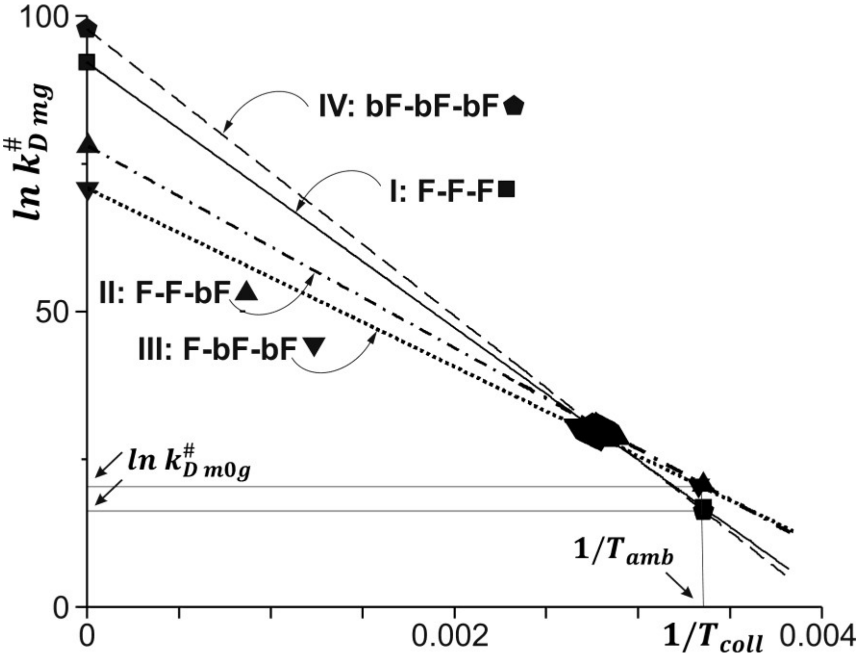

Supplemental Figure S7: Arrhenius plot for (biotinylated) T4Ff trimer dissociation reactions in the gas phase. T4Ff homo-trimer: ■; singly biotinylated T4Ff hetero-trimer: ▲; doubly biotinylated T4Ff hetero-trimer: ▼; biotinylated T4Ff homo-trimer: ⬟. Each data point (thickened parts of the lines) has been obtained experimentally and corresponding lines have been linearly extrapolated. The values for $ln\ \mathrm{k}^{\#}_{\mathrm{D\ m0g}}$ are taken at $1/_{\mathrm{T_{amb}}}$ (cf. Table 3).

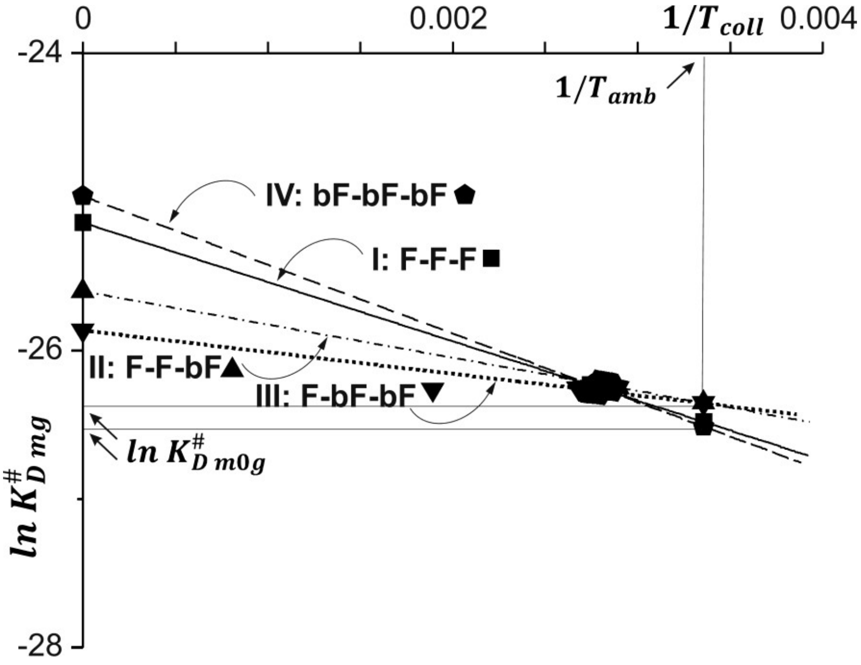

Supplemental Figure S8: Gibbs-Helmholtz plot for (biotinylated) T4Ff trimer dissociation reactions in the gas phase. T4Ff homo-trimer: ■; singly biotinylated T4Ff hetero-trimer: ▲; doubly biotinylated T4Ff hetero-trimer: ▼; biotinylated T4Ff homo-trimer: ⬟. Each data point (thickened parts of the lines) has been obtained experimentally and corresponding lines have been linearly extrapolated. The values for $ln\ \mathrm{k}^{\#}_{\mathrm{D\ m0g}}$ are taken at $1/_{\mathrm{T_{amb}}}$ (cf. Table 3).

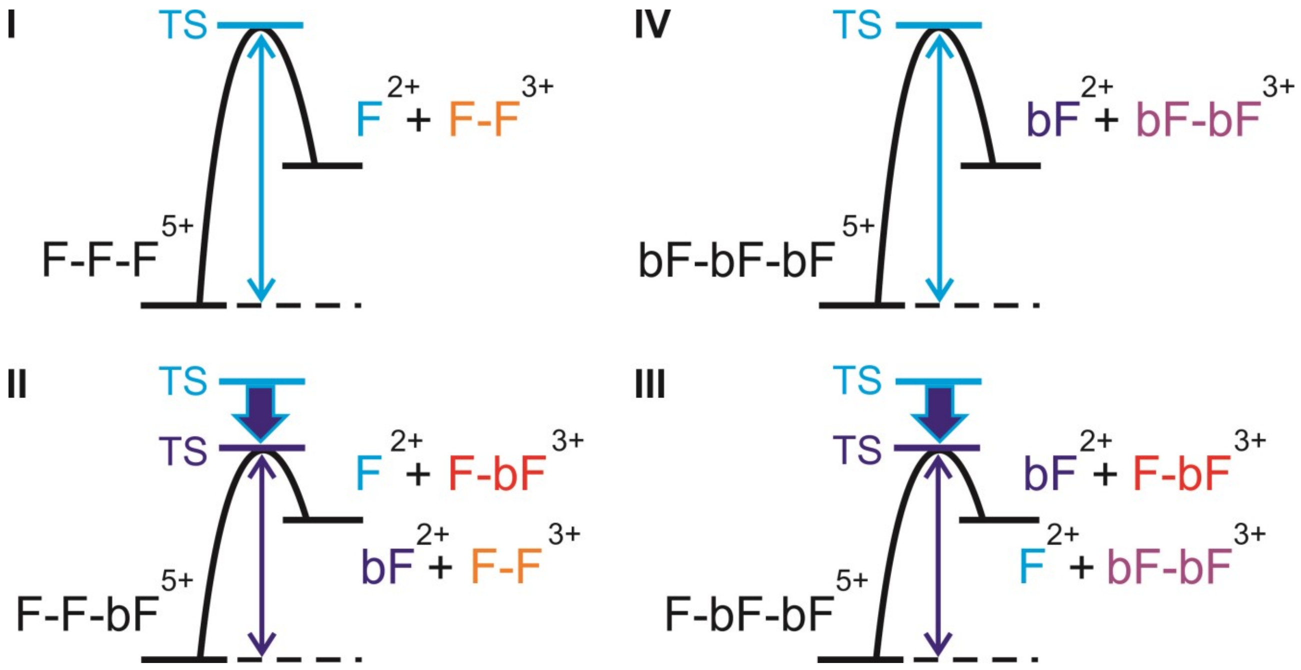

Supplemental Figure S9: Energy diagrams showing the apparent enthalpies of activation required by multiply charged and accelerated (biotinylated) T4Ff trimers ($\Delta H^{\#}_{m0g}$) to reach the transition state (TS) before dissociating into products ions. **I:** T4Ff homo-trimer (F-F-F). **II:** singly biotinylated T4Ff hetero-trimer (F-F-bF). **III:** doubly biotinylated T4Ff hetero-trimer (F-bF-bF). **IV:** biotinylated T4Ff homo-trimer (bF-bF-bF). The dark blue arrow indicates lowering of the energy level of the transition states of the hetero-trimers with respect to those of the homo-trimers.
